# Determination of demographic, epidemiological, and socio-economic determinants and their potential impact on malaria transmission in Mannar and Trincomalee districts of Sri Lanka

**DOI:** 10.1186/s12936-016-1390-7

**Published:** 2016-06-22

**Authors:** Nayana Gunathilaka, Wimaladharma Abeyewickreme, Menaka Hapugoda, Rajitha Wickremasinghe

**Affiliations:** Department of Parasitology, Faculty of Medicine, University of Kelaniya, Colombo, Sri Lanka; Molecular Medicine Unit, Faculty of Medicine, University of Kelaniya, Colombo, Sri Lanka; Department of Public Health, Faculty of Medicine, University of Kelaniya, Colombo, Sri Lanka

**Keywords:** Demographic, Epidemiological, Socio-economic, Malaria

## Abstract

**Background:**

Malaria was an endemic problem in Mannar and Trincomalee districts of Sri Lanka until the recent past. Currently, no local case has been found since October 2012. Therefore, the present study was conducted to identify existing demographic, epidemiological and socio-cultural factors in Mannar and Trincomalee districts of Sri Lanka, since there is limited information available on the potential influence of above variables responsible for low malaria transmission.

**Methods:**

An analytical cross-sectional survey was carried out on selected demographic, epidemiological and socio-economic variables in 32 localities under eight sentinel sites (Each sentinel with four localities) using a pre-defined questionnaire during June–September 2012. Household heads of 45 houses from each locality were selected randomly to participate in the present study. Data were analysed using the Paired Chi Square test and Bray–Curtis method.

**Results:**

A total of 1440 household heads were interviewed. Both districts indicated statistically acceptable similarities (p > 0.05) in age structure, gender, family size and presence of animals. The knowledge on malaria was observed under “Poor” category. The protective measures against mosquito bites, spraying status of houses and occupation pattern were varied significantly in both districts (p < 0.05). Educational level was statistically similar (p > 0.05) in both districts. Majority of the families were identified as living in “Moderate” house type under low economic condition. Both populations were indicated 85 % similarity according to Bray–Curtis analysis.

**Conclusion:**

Lack of awareness in these communities about the disease may facilitate to the re-emerge of malaria.

## Background

Malaria is a vector borne disease transmitted between humans by adult female mosquitoes of the genus *Anopheles*. The disease is endemic in tropical and subtropical regions of the world [1]. Malaria was a major public health problem in Sri Lanka in the past. The most prevalent cause of malaria was *Plasmodium vivax* (70 %) while the rest of the cases were caused by *Plasmodiumfalciparum* [2]. Sri Lanka has not reported a local case of malaria since October 2012. This remarkable success was achieved rapidly and largely during a protracted civil war. As recently as 2000, Sri Lanka had over 100,000 cases of malaria each year [3].

Several factors have impacted on low malaria transmission during last two decades [4]. The favourable factors include increased reliance on self-protection methods, early detection and treatment of patients by mobile malaria clinics. In addition, changes in socio-economic and living standards in the community may have a positive impact on reducing malaria cases at present.

Mannar and Trincomalee districts were previously considered as highly malarious in northern and eastern provinces of Sri Lanka, respectively. As a result of resettlement and development activities there have been many changes in living standards among communities in these areas. Therefore, the objective of the present study was to determine how existing epidemiological, demographic and socio-economic factors contributed to have zero indigenous malaria and their potential impact on malaria transmission in Mannar and Trincomalee districts of Sri Lanka.

## Methods

### Study area

**Fig. 1:**
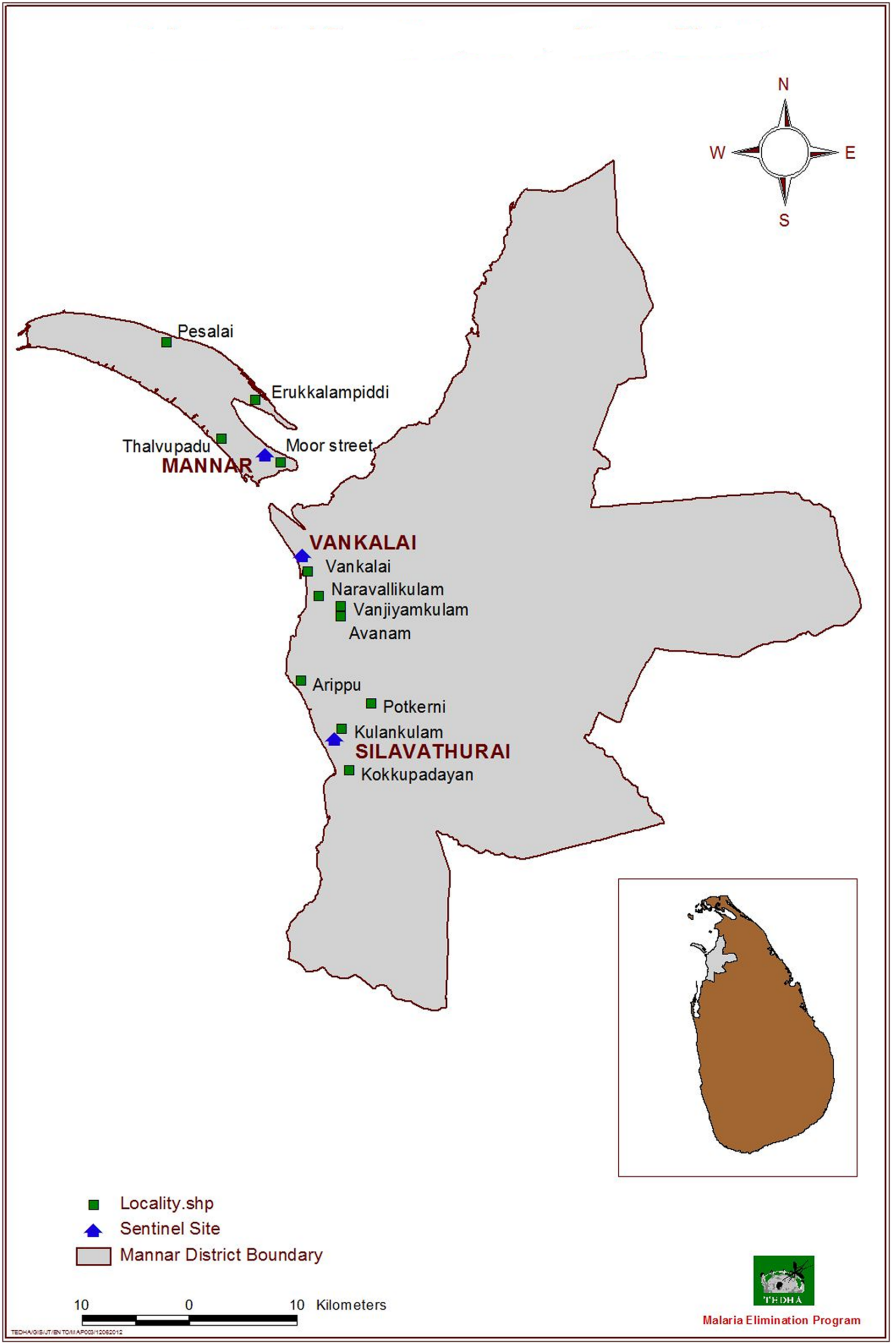
Sentinel sites and localities selected for the surveys in Mannar District

**Fig. 2:**
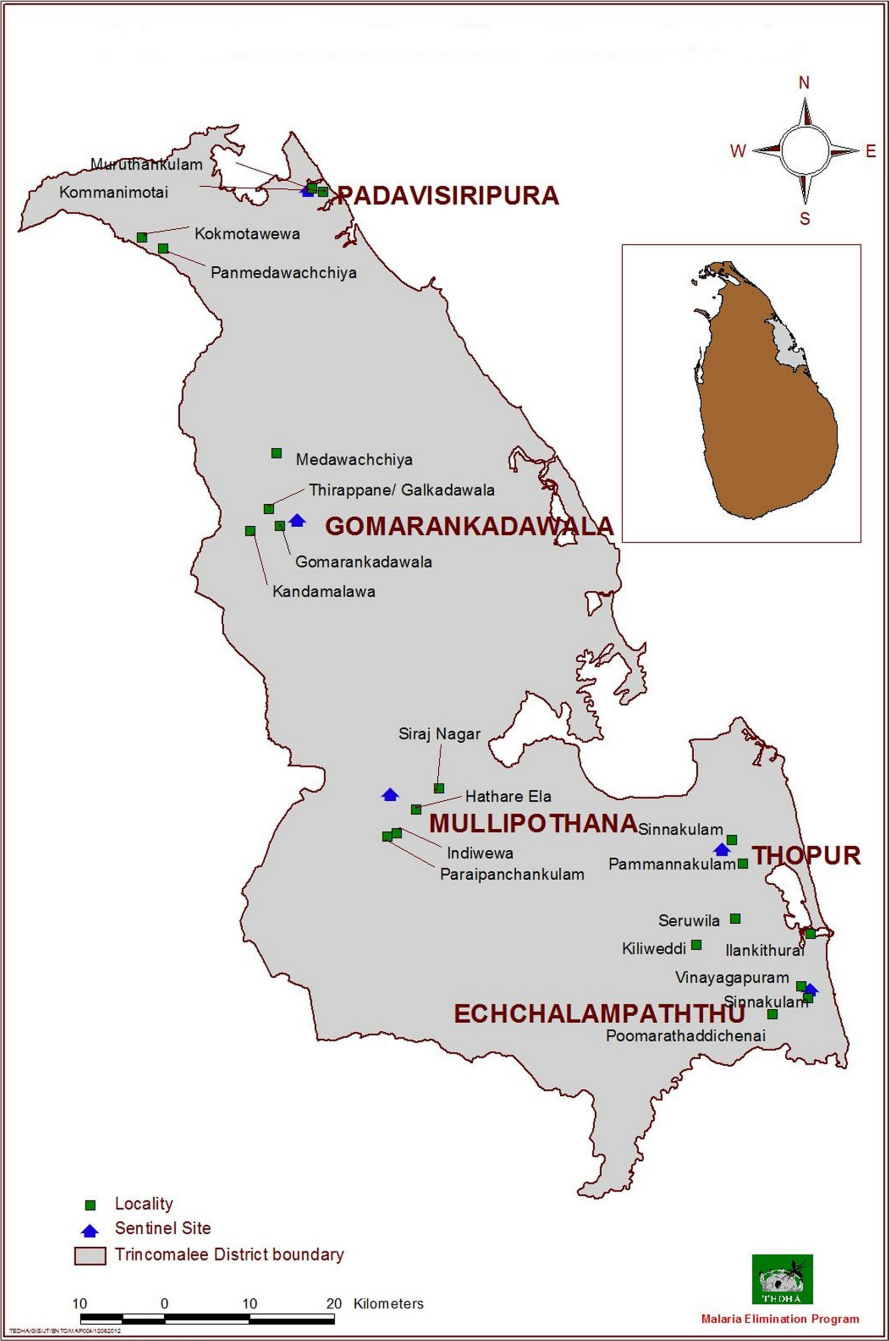
Sentinel sites and localities selected for the surveys in Trincomalee District

Mannar (08°52′N, 80°04′E) and Trincomalee (08°35′N, 81°05′E) districts are located in the northern and eastern provinces of Sri Lanka which are having an area of 1996 and 2727 km^2^, respectively. A total of eight sentinel sites, three in the district of Mannar (Fig. 1) and five in the district of Trincomalee (Fig. 2) were selected. Each malaria sensitive sentinel site having a radius of 20 km was further subdivided into four localities (within 5–20 km) to ensure full coverage of the sentinel site during the surveillance. Hence, a total of 32 localities in eight sentinel sites were identified and each locality was given a name code. Selection of the localities was based on past malaria history, environmental conditions, availability of breeding sites, an established agricultural community and feasibility of field operations to collect relevant data.

### Study design

This analytical cross-sectional survey was carried out from June–September 2012. Two hundred houses were identified at each locality. Of them 45 houses from each locality were selected randomly to participate in the present study.

During the surveys, about 540 and 900 household heads covering 2455 and 3859 individual household members were interviewed in Mannar and Trincomalee districts, respectively. The head of the household was defined as the person who perceived by household members to be the primary decision maker in the family and the household was defined as individuals living together and taking meals from a common cooking facility. In the absence of a household head, a responsible adult above 18 years who appointed by the family was interviewed.

### Data collection

Data on demographic (age, gender, family size, presence of domestic animals), epidemiological (knowledge/awareness on malaria, potential breeding sites around houses/proximity, use of protective measures against mosquito bite, past malaria history, insecticide spraying status of the house) and socio-economic (level of education, monthly income, house type, occupation pattern of the head of household) determinants among two study populations were obtained using a structured questionnaire that was printed in English and local languages (Sinhala and Tamil).

### Data analysis

Double data entry was done using Microsoft access for validation purpose before analysis. The epidemiological, demographic and socio-economic factors which may potentially influence on malaria transmission in the districts of Mannar and Trincomalee were statistically compared by using the Paired Chi Square test. The existing factors were compared with the previously recognized risk factors of malaria transmission in accordance with the already published literature. In addition, Bray–Curtis similarity clustering method (with square root transformation) was devised to investigate similarities among two study populations.

## Results

### Demographic characteristics

The age group of 16–50 years was predominant both in Mannar and Trincomalee districts. Male to female ratio of both populations were approximately equal among all age groups, without any significant differences as suggested by the Chi square test (p > 0.05, at 95 % level of confidence). Both communities were shared statistical similarities among age and gender structures (p > 0.05). The families with 4–6 members were identified as the predominant family type in both districts followed by 1–3 and >7 per family (Table 1). When considering the presence of animals, hens were the widely reared animal among two study populations. Overall, both districts indicated statistically acceptable similarities (p > 0.05) in terms of age structure, gender, average family size and presence of animals (Table 1).

**Table 1:** Demographic characteristics and their association with malaria transmission among the study population in Mannar and Trincomalee Districts

Characteristics	Mannar		Trincomalee		P	*X* ^*2*^
	N	%	N	%		
Age					0.96	0.58
<1	31	1.26	79	2.05		
1–5	239	9.74	416	10.78		
6–15	594	24.20	884	22.91		
16–50	1285	52.34	2095	54.29		
>50	306	12.46	385	9.98		
Gender					0.83	0.04
Male	1260	51.53	1932	50.06		
Female	1185	48.47	1927	49.94		
Family size					0.43	1.67
1–3	131	24.26	291	32.33		
4–6	353	65.37	536	59.56		
>7	56	10.37	74	8.22		
Presence of animals					0.14	8.18
Cattle	290	8.16	866	17.22		
Goat	91	2.56	168	3.34		
Dog	195	5.48	531	10.56		
Cat	132	3.71	337	6.70		
Hen	2820	79.30	3076	61.15		
Other	28	0.79	52	1.03		

p < 0.05 indicates a significant difference among the two populations

### Epidemiological characteristics

Epidemiological determinants among two study copulations are illustrated in Table 2. The knowledge on malaria among study populations in Mannar (52.04 % n = 281) and Trincomalee (64.33 %, n = 579) was characterized under “Poor” category. The majority of households were remained with no potential breeding sites. Only 0.3 % (n = 7) and 0.1 % (n = 4) from Mannar and Trincomalee were experienced at least a single attack of malaria during their lifetime respectively.

**Table 2:** Malaria epidemiology related characteristics of the study population in Mannar and Trincomalee Districts

Characteristics	Mannar		Trincomalee		p	*X* ^*2*^
	N	%	N	%		
Knowledge about malaria					0.09	4.69
Poor	281	52.04	579	64.33		
Adequate	192	35.56	196	21.78		
Good	67	12.41	125	13.89		
Potential breeding sites					0.29	7.26
Burrow pit	37	6.85	79	8.78		
Earth well	23	4.26	105	11.67		
Built well	152	28.15	171	19.00		
Tank/pond	80	14.81	144	16.00		
Paddy field	48	8.89	116	12.89		
Cemented tank	12	2.22	32	3.56		
None	188	34.81	253	28.11		
Use of protective measures					0.01	16.63
Residual spraying	36	6.67	8	0.89		
Bed net	402	74.44	534	59.33		
Covering eve and window	16	2.96	39	4.33		
Mosquito coil	16	2.96	123	13.67		
Integrated approach	58	10.74	143	15.89		
Other	1	0.19	23	2.56		
None	11	2.04	30	3.33		
Past malaria infection					0.51	0.42
Yes	7	1.30	4	0.44		
No	533	98.70	896	99.56		
Spraying status of the house					0.00	85.41
Sprayed	96	17.78	54	6.00		
Overdue	106	19.63	767	85.22		
Unsprayed	338	62.59	88	9.78		

p < 0.05 indicates a significant difference among the two populations

The protective measures against mosquito bites by two populations were significantly different (p < 0.05 at 5 % level of significance). The use of bed net was the predominant practice used against mosquito bites. Among all families, the average number of bed nets per family was 2.04. Some individuals were not used at least on protective measure among the study population in Mannar (2.04 %, n = 11) and Trincomalee (3.3 %, n = 30) districts. There was a significant difference of the spraying status of houses among both study communities (p < 0.05).

### Socio-economic characteristics

As suggested by the Paired Chi Square test, two populations were statistically similar in educational level (p > 0.05 at 5 % level of significance). Individuals with secondary education (Grade 6–11) were observed as the highest in Mannar (51.85 %, n = 1231) and Trincomalee (50.84, n = 1962).

**Fig. 3:**
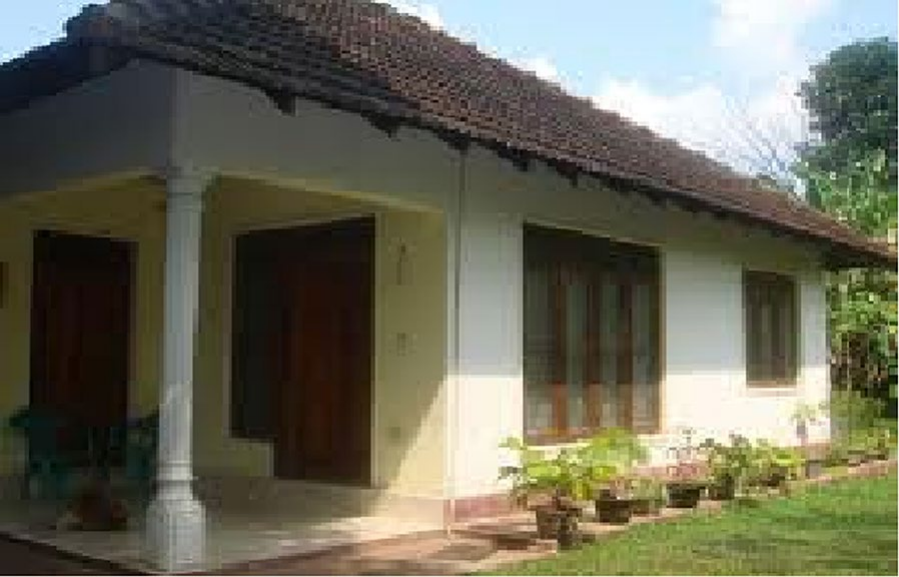
Example of a “Good” house

**Fig. 4:**
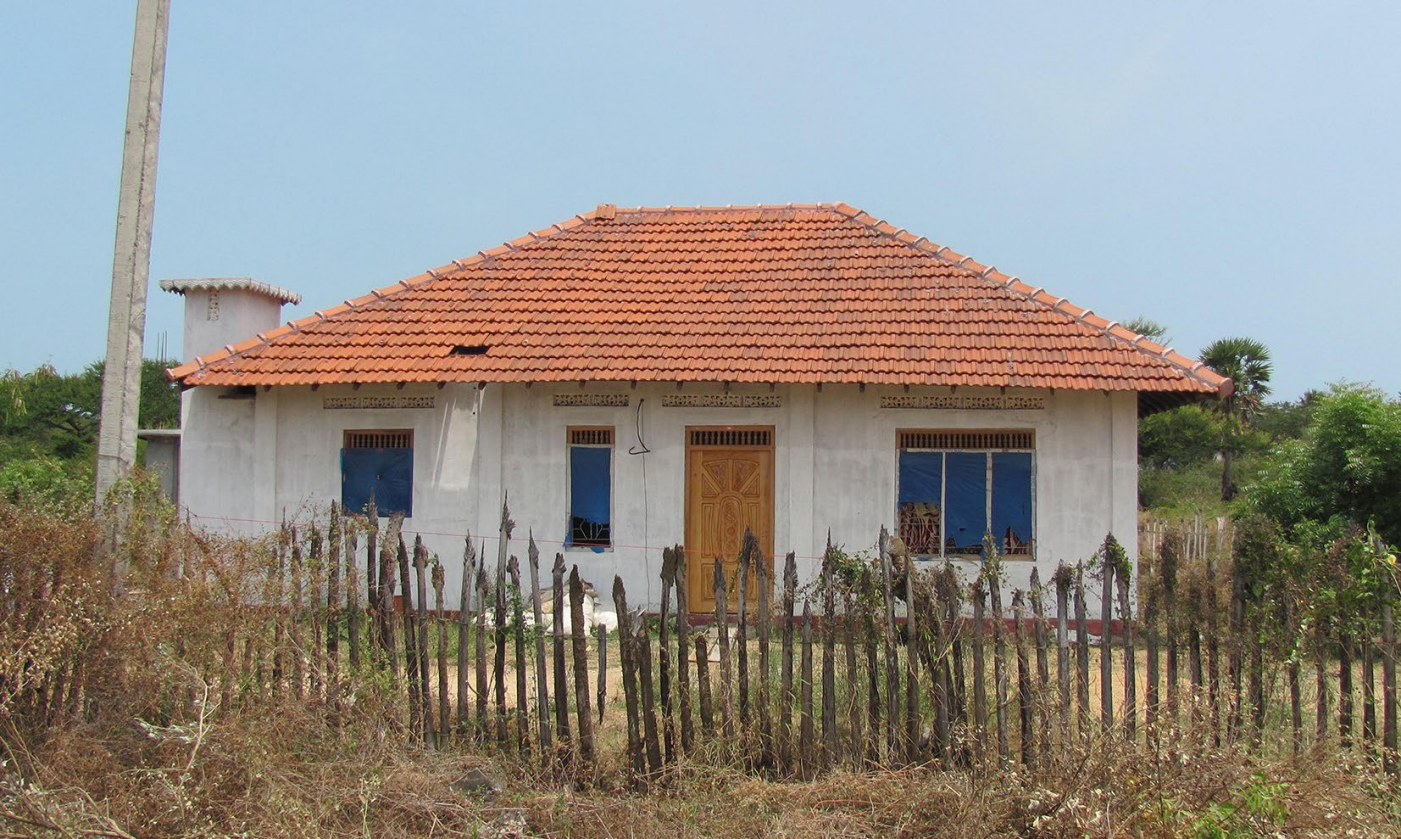
Example of a “Moderate” house

**Fig. 5:**
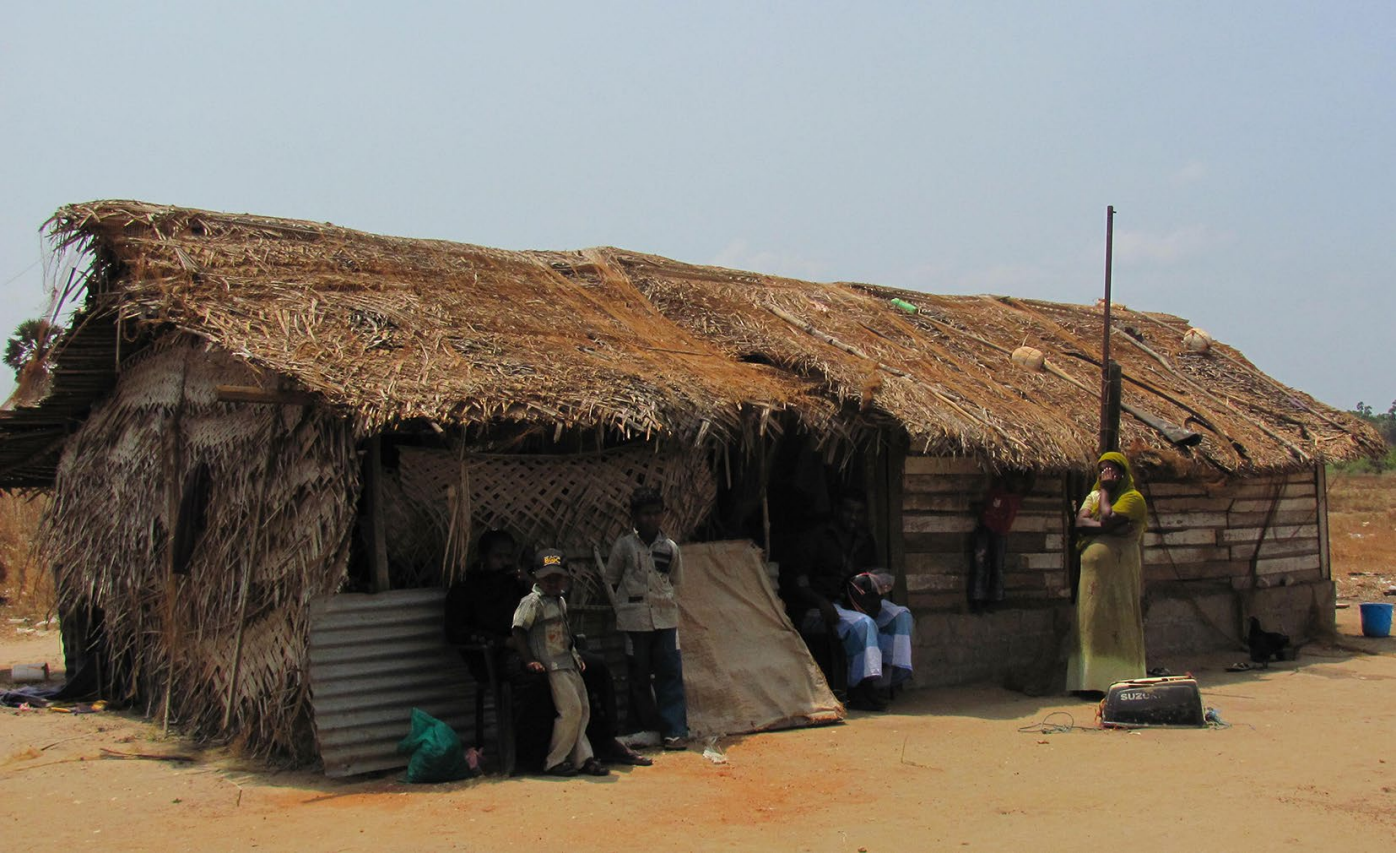
Example of a “Poor” house

The mean monthly income was ranged from 5001 to 10,000 Sri Lankan rupees (Rs) (459.26–918.34 USD). The houses having plastered cement walls with tiled or asbestos roofs were categorized as “Good” (Fig. 3) while un-plastered brick walls with tiled or asbestos roofs were considered as “Moderate” (Fig. 4). All other types were grouped as “Poor” houses (Fig. 5). Of these three categories, the “Moderate” house type was predominant in both Mannar (42.22 %) and Trincomalee (56.56 %) districts (Table 3). The occupation types of heads of households among two study populations were varied significantly (p < 0.05 at 5 % level of significance).

**Table 3:** Socio-economic characteristics of the study population in Mannar and Trincomalee Districts

Characteristics	Mannar		Trincomalee		p	*X* ^*2*^
	N	%	N	%		
Education level					0.59	4.63
Bellow school age	236	9.94	495	12.83		
1–5	427	17.99	810	20.99		
6–11	1231	51.85	1962	50.84		
12–13	321	13.52	237	6.14		
Diploma	4	0.17	3	0.08		
Degree	24	1.01	10	0.26		
None	131	5.52	342	8.86		
Monthly income in Sri Lankan Rupee					0.43	3.79
≤5000	202	37.41	266	29.56		
5001–10,000	232	42.96	372	41.33		
10,001–20,000	72	13.33	207	23.00		
20,001–30,000	18	3.33	37	4.11		
>30,000	16	2.96	18	2.00		
House type					0.12	4.11
Good	112	20.74	143	15.89		
Poor	200	37.04	248	27.56		
Moderate	228	42.22	509	56.56		
Occupation of the head of the household					0.003	31.11
Army/forces	1	0.19	127	14.11		
Carpenter	6	1.11	7	0.78		
Driver/conductor	11	2.04	12	1.33		
Farmer	127	23.52	363	40.33		
Fisherman	120	22.22	78	8.67		
Foreign employment	1	0.19	11	1.22		
Government servant	41	7.59	48	5.33		
Labourer	132	24.44	106	11.78		
Mason	3	0.56	16	1.78		
Mechanic	12	2.22	22	2.44		
Other	45	8.33	66	7.33		
Self-employed	6	1.11	4	0.44		
Traders/business	35	6.48	40	4.44		

p < 0.05 indicates a significant difference among the two populations

### Bray–Curtis similarity clustering analysis

**Fig. 6:**
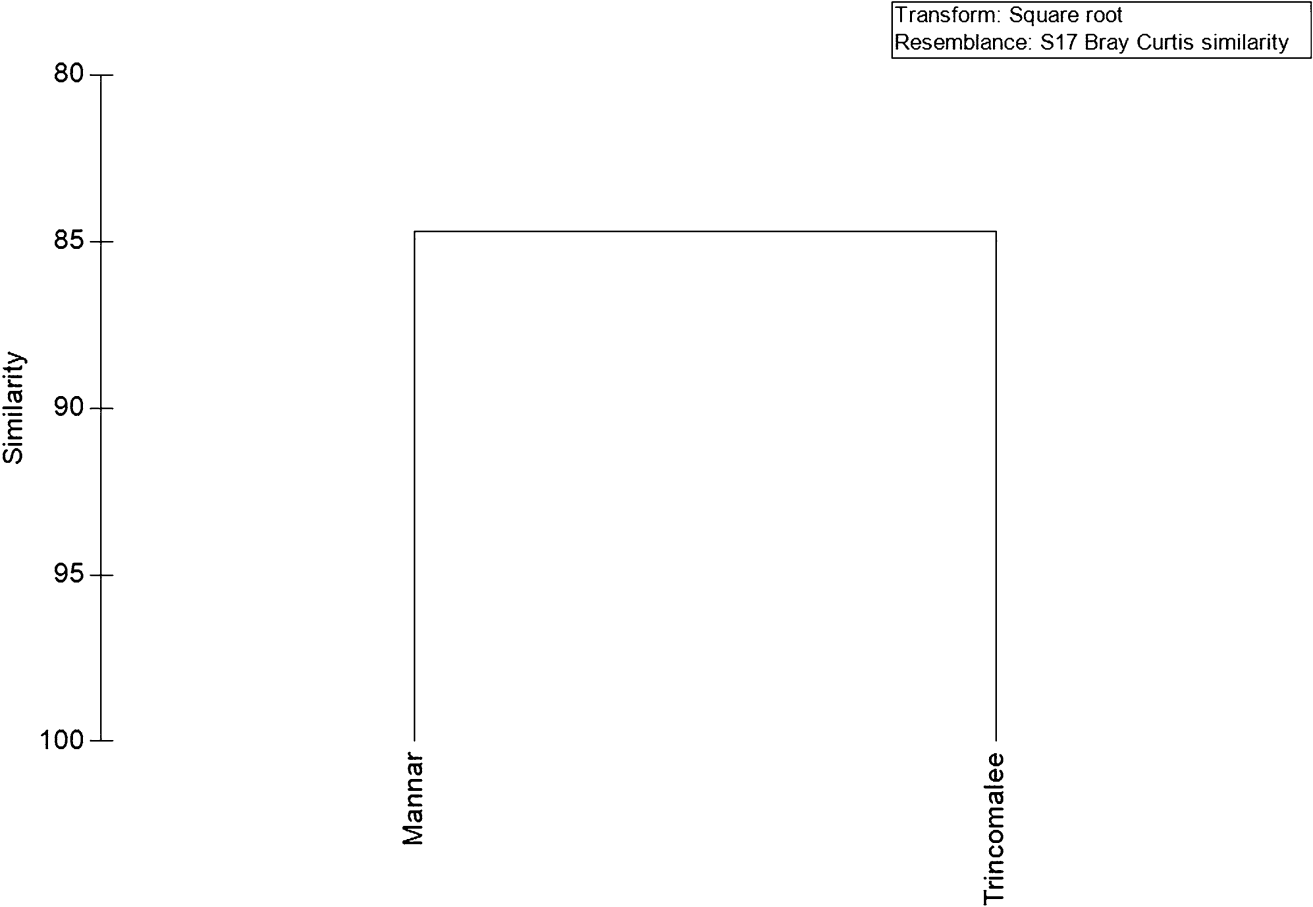
The *dendogram* for the cluster analysis of demographic, epidemiological and socio-economic characteristics in two study populations

The overall clustering status of two populations in terms of demographic, epidemiological and socio-economic characteristics is illustrated in Fig. 6. As suggested by the Bray–Curtis similarity, the two populations in the districts of Mannar and Trincomalee were shared a similarity of 85 % (Fig. 6). The dissimilarity level of 15 % among two populations could be associated with some significant differences in protective measures, spraying status and occupation status of household heads.

## Discussion

The present study was carried out in Mannar and Trincomalee districts of Sri Lanka, which were considered as high-risk areas for malaria infections previously, in order to determine demographic, epidemiological and socio-economic determinants on potential malaria transmission and how these factors contributed to low malaria prevalence in the study districts. However, correlations between prevalence of malaria cases and potential risk factors were not able to carry out due to no indigenous cases at present in both districts.

### Demographic characteristics

Demographic and geographical factors such as gender, age, family size and the region where the people live may effect on the risk of malaria transmission [5]. It was observed that the positive malaria diagnostic rate decreases with age and risk of malaria increased per unit increase in family size. Generally, malaria parasite prevalence differed between age and gender with the highest prevalence in children and females. However, only few of them had past malaria infections among the study populations in Mannar (1.3 %) and Trincomalee (0.44 %). Therefore, no positive correlation was identified in terms of gender, age, family size or region with malaria infection.

From the climatic point of view, the study regions are conducive to malaria epidemics and can be supported by the presence of malaria vectors, *Anopheles culicifacies, Anopheles subpictus, Anopheles annularis, Anopheles varuna* and *Anopheles tessellatus* [6–8]. These areas have been on a steep development trajectory after the end of separatist war in terms of building houses, urban development, road constructions and rapidly growing tourist industry, all of which are associated with increased travel of foreign nationals; and introduction of foreign labour into the country, increasing the number of imported malaria cases [9]. Ongoing construction projects are leading to the creation of new vector breeding sites, including in previously endemic areas. Recent findings identified a conducive breeding of malaria vectors including *An. culicifacies* sibling species E in waste water containing drains in urban/semi-urban settings [10]. Therefore, changes in the demography have increased the receptivity to malaria in previously endemic areas.

Many of the houses kept animals in their households namely; cattle, goats, dogs, cats and poultry. Since malaria vectors are zoophilic in nature, there is a possibility of attracting vectors to animals. Therefore, the absence of malaria incidence in these study areas may be due to the phenomenon of zooprophylaxis [11]. Some recent studies conducted to determine foraging behaviour of malaria vectors in the country also suggest that human is not the preferred host for tested anophelines [3].

### Epidemiological characteristics

Socio-economic status and human settlement patterns also affect human vulnerability to vector-borne diseases. For example, if preventive measures such as screens, insect repellants or other practices are available and affordable to risk populations of mosquito-borne diseases, infection can be drastically lowered [12].

The present case study identified residual spraying, use of bed nets (LLIN, ITN or normal nets), covering eaves/windows and use of mosquito coils as the main preventive measures against mosquito biting. About 10.74 and 15.89 % in the districts of Mannar and Trincomalee showed integrated vector control methods, which contained at least two preventive measures. In addition, only 0.18 and 2.46 % of the total families surveyed in the districts of Mannar and Trincomalee indicated the use of traditional methods such as creating a smoke with “Maduruthala” (*Ocimum sanctum*) leaves or coconut shells, applying citronella oil on the skin, which categorized as other preventive measures against mosquito biting. Hence, increasing the use of preventive measures against vector biting has caused a negative impact on the malaria transmission.

The knowledge and awareness has a significant influence on malaria control Individual knowledge, awareness and beliefs may also affect malaria occurrence [13, 14]. However, the awareness of community on malaria was poor. Severity of the disease was also not known by 75 % of the total population surveyed. This may be due to the absence of indigenous cases in the country at present. However, this situation may facilitate to re-emerge the disease as a result of poor awareness about the disease by the community, since the lack of adequate knowledge among the general public about the disease and its prevention is an important contributory factor for the disease transmission [15].

### Socio-economic characteristics

Malaria is considered as a disease associated low income communities with poor socio-economic status because, poor communities have comparatively less access to anti-malarials and anti-mosquito measures, since they cannot afford personal protection measures, a clean environment free of mosquito breeding sites, vulnerable to ineffective diagnosis and treatment due to financial and cultural implications [5, 16]. Therefore, a better understanding of the relationships between malaria and socio-economic variables is needed to enable design effective policies and tools to tackle the problem.

The present study revealed that the majorty of these communities were in the middle economic strata lived under “moderate” housing condition. Previous studies conducted in southern Sri Lanka have shown that over 70 % of the malarial episodes were reported from “Poor” houses [12, 17]. It was further explained that the “Poor” house type with mud, cadjan or brick un-plastered walls with thatched or asbestos roofs was the most predominant house type in that study. It is most likely that poorly constructed mud cadjan houses might have a number of gaps and holes through which a vector mosquito could easily enter following the scent of human hosts. However, in the present study, “Good” and “Moderate” house types were higher than of “Poor” house type having mud or cadjan wall with cadjan roof, which does not support malaria vector mosquitoes to rest inside houses. Therefore, bricks and cement walls may limit the contact with mosquito vector also reduces the possibility of being bitten by a vector mosquito [5]. Hence, changing living style in these areas may have a negative impact on malaria transmission.

The predominant average monthly income category in both districts was of Rs. 5001–10,000. It is indicative that both districts represented a low economic condition which should be lead to increase “Poor” house type. However, this situation can be explained that due to the availability of resources in these areas have been facilitated them to build or renovate houses to “Moderate” or “Good” conditions and some development activities which were initiated by the government/non-governmental organizations have been significantly influenced on uplifting the house types.

Sri Lanka is an agricultural country in which the majority of the country’s population engages in agricultural related activities and lives in traditional malarious areas [17]. The peak of malaria transmission in the county coincided between important agricultural activities during the past. Out of 12 main occupation categories, only 23.54 % from Mannar and 40.33 % from Trincomalee represented “Farmer”, which is significantly low when compared to the previous studies conducted in Sri Lanka [17–19]. It seems that majority of the people living in the districts of Mannar and Trincomalee do not engage with direct agricultural activities.

The agricultural activity practices by farmers were paddy cultivation, which dominated by land preparation followed by planting of crops, application of fertilizer, application of agro-chemicals for the control of pest/weeds and harvesting the crops. In early days, people were used to follow traditional, time consuming practices for land preparation and cultivation till late night. However, as a result of expanding commercial agriculture and use of mechanical equipment have reduced the time consume for agricultural activities. Therefore, number of days stayed outside for ploughing and harvesting or time stays out-door till late evening have been reduced significantly. Hence, the people get less chances of exposing to mosquito bites, which would make them less susceptible to malaria.

Overall, some established risk factors in this study were not associated with malaria transmission. However, current study on potential risk factors affecting transmission of malaria is important for a country which is achieving elimination and entering into prevention of reintroduction phase. Therefore, it is essential to keep the people aware on malaria and especially some people in these districts visit nearby countries like India and Pakistan where still suffering from the disease severely. Hence, these people could be vulnerable to malaria at their visit any time and even from them a reintroduction of malaria could be occurred in the country due to imported cases with the presence of malaria vectors in these areas.

## Conclusion

Improvement in living standard and commercial agriculture were identified as potential factors associated with the reduction of malaria incidence. However, lack of awareness in these communities about the disease may facilitate to re-emerge the malaria incidence. Therefore, these findings would be helpful in better planning and implementation of malaria control strategies in the study areas.

## Abbreviations

Rs: Sri Lankan rupees
LLIN: long-lasting insecticide-treated bed net
ITN: insecticide-treated bed net
